# Warm Ischemia Induces Spatiotemporal Changes in Lysophosphatidylinositol That Affect Post-Reperfusion Injury in Normal and Steatotic Rat Livers

**DOI:** 10.3390/jcm12093163

**Published:** 2023-04-27

**Authors:** Kengo Shibata, Takahiro Hayasaka, Sodai Sakamoto, Satsuki Hashimoto, Norio Kawamura, Masato Fujiyoshi, Taichi Kimura, Tsuyoshi Shimamura, Moto Fukai, Akinobu Taketomi

**Affiliations:** 1 Department of Gastroenterological Surgery I, Graduate School of Medicine, Hokkaido University, Sapporo 060-8638, Japan; 2 Department of Transplant Surgery, Graduate School of Medicine, Hokkaido University, Sapporo 060-8638, Japan; 3 Department of Cancer Pathology, Faculty of Medicine, Hokkaido University, Sapporo 060-8638, Japan; 4 Division of Organ Transplantation, Hokkaido University Hospital, Sapporo 060-8648, Japan

**Keywords:** imaging mass spectrometry, warm ischemia and reperfusion injury, lysophosphatidylinositol

## Abstract

Warm ischemia-reperfusion injury is a prognostic factor for hepatectomy and liver transplantation. However, its underlying molecular mechanisms are unknown. This study aimed to elucidate these mechanisms and identify the predictive markers of post-reperfusion injury. Rats with normal livers were subjected to 70% hepatic warm ischemia for 15, 30, or 90 min, while those with steatotic livers were subjected to 70% hepatic warm ischemia for only 30 min. The liver and blood were sampled at the end of ischemia and 1, 6, and 24 h after reperfusion. The serum alanine aminotransferase (ALT) activity, Suzuki injury scores, and lipid peroxidation (LPO) products were evaluated. The ALT activity and Suzuki scores increased with ischemic duration and peaked at 1 and 6 h after reperfusion, respectively. Steatotic livers subjected to 30 min ischemia and normal livers subjected to 90 min ischemia showed comparable injury. A similar trend was observed for LPO products. Imaging mass spectrometry of normal livers revealed an increase in lysophosphatidylinositol (LPI (18:0)) and a concomitant decrease in phosphatidylinositol (PI (18:0/20:4)) in Zone 1 (central venous region) with increasing ischemic duration; they returned to their basal values after reperfusion. Similar changes were observed in steatotic livers. Hepatic warm ischemia time-dependent acceleration of PI (18:0/20:4) to LPI (18:0) conversion occurs initially in Zone 1 and is more pronounced in fatty livers. Thus, the LPI (18:0)/PI (18:0/20:4) ratio is a potential predictor of post-reperfusion injury.

## 1. Introduction

Warm ischemia-reperfusion injury (WIRI) is one of the factors that determine the outcomes of liver surgery [1]. In liver transplantation, non-standard grafts (such as grafts from donation after cardiac death (DCD) and fatty liver grafts) have been explored to expand the donor pool [2]. In liver transplantation using DCD grafts, WIRI progresses in three stages: (1) warm ischemia (hypoxia) due to cardiac arrest, (2) subsequent cold ischemia (hypothermia and hypoxia), and (3) reperfusion (re-warming and reoxygenation) [3]. Therefore, DCD grafts are mainly classified by the duration of warm ischemia [4]. The safe use of uncontrolled DCD grafts is a recognized goal of basic and clinical research [5]. In hepatectomy, the Pringle maneuver is a standard technique to reduce bleeding [6]; however, the remnant liver is damaged by warm ischemic insult. Recently, the number of hepatectomies involving fatty livers has been increasing due to an increase in the number of patients with nonalcoholic fatty liver disease (NAFLD). Therefore, it is imperative to reduce WIRI in fatty livers [7].

WIRI progresses through reactive oxygen species (ROS) and inflammatory cytokines mainly released from Kupffer cells, which lead to direct hepatocyte injury, expression of adhesion molecules on the surface of sinusoidal endothelial cells, neutrophil chemotaxis, and exacerbated inflammation [8]. Concomitant oxidative stress causes cell injury via oxidative modification of lipids, proteins, and nucleic acids [9]. In a fatty liver, accumulated fatty acids and phospholipids enhance inflammatory reactions, leading to an impaired mitochondrial function [10], reduced adenosine triphosphate (ATP) synthesis, and accelerated ROS production [11]. Therefore, a comprehensive understanding of lipids and cellular function is necessary to elucidate the pathophysiology and underlying mechanisms of WIRI in a fatty liver.

Changes in free fatty acid content and extensive lipid peroxidation (LPO) during warm ischemia result in hepatic injury after reperfusion [12]. To overcome this problem, our research group has been focusing on reducing cell injury caused by microcirculatory disturbance. Administration of antioxidants and Rho kinase (ROCK) inhibitors [13] and administration of hydrogen during reperfusion [14] can improve microcirculation and reduce injury. Notably, LPO progresses during warm ischemia and causes irreversible post-reperfusion injury. However, the precise structure of the LPO product remains unknown. Thus, we hypothesized that low-molecular-weight compounds, including lipids and fatty acids, cause and accelerate hepatic injury during warm ischemia and after reperfusion. To test this hypothesis, in the present study, we explored the temporal and spatial variations in low-molecular-weight compounds using imaging mass spectrometry (IMS).

IMS acquires the mass spectra of ionized molecules by laser irradiation of frozen sections. MS is based on the ionization of a sample after which the resulting ions are identified, classified, and analyzed qualitatively and quantitatively. Matrix-assisted laser desorption/ionization (MALDI) is based on IMS; a matrix is applied to the sample, and a laser beam is then applied to all areas of the sample [15]. 

**Figure 1:**
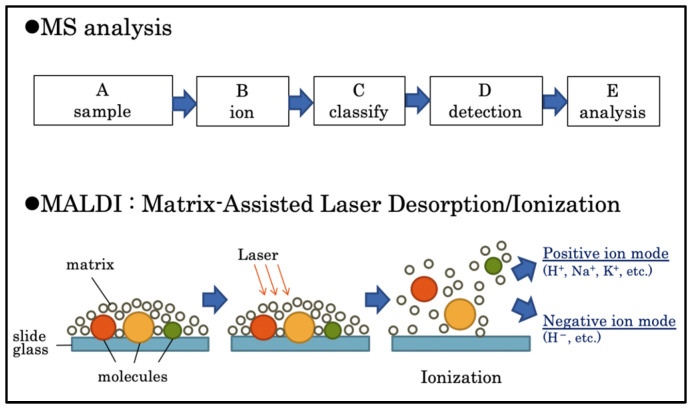
Principle of mass spectrometry. Mass spectrometry analyzes samples qualitatively and quantitatively through ionization, classification, identification, and analysis (in that order). In matrix-assisted laser desorption/ionization, a matrix is applied to the sample; the matrix is then rapidly heated by a laser beam and vaporized and ionized with the sample.

The MS spectrum of each region is repeated to obtain the MS data for the entire section (Figure 1). The signal intensities, detected as different mass-to-charge ratios (*m*/*z*), are displayed as pseudo colors in each pixel to visualize the distribution and quantity of the substance [16]. Recent studies have used IMS for analyzing lipid distribution [17] in rat brains during ischemia-reperfusion [18], pathological analysis of the relationship between lipid regulation and congenital valvular disease in human heart valves [19], pharmacokinetic analysis, and evaluation of therapeutic effects [20].

The present study aimed to elucidate the mechanism of WIRI in normal and steatotic rat livers and identify the predictive markers of post-reperfusion injury by performing a comprehensive IMS-based analysis of the temporal and spatial changes in small molecule compounds.

## 2. Methods

Reagents that are not listed were purchased from Fujifilm Wako Pure Chemicals Co. (Osaka, Japan).

### 2.1. Study Design and Animals

All animal experiments were performed in accordance with the “Regulations for Animal Experiments at Hokkaido University” and with the approval of the Hokkaido University Animal Experiment Ethics Committee (no. 17-0032). This study was performed on 9-week-old male Wistar rats (Nihon SLC Co. Oriental Yeast Industry Co., Ltd., Tokyo, Japan) that were acclimatized for 1 week after admission and housed in a steady-state environment (23 ± 2 °C, 50 ± 10% humidity, and 12 h light/dark cycle).

### 2.2. Fatty Liver Model

The rats were fed a standard diet for three days after admission; thereafter, they were kept fasting for two days and then fed a high-carbohydrate diet (HCD; Research Diets Inc., New Brunswick, NJ, USA) for three days. The HCD comprised 85% carbohydrates (50% sucrose and 50% corn starch), 15% protein, and 0% fat. Fatty liver was evaluated using the NAFLD Activity Score (NAS), which is a sum of the scores for steatosis (S; 0: <5%, 1: 5–33%, 2: 33–66%, and 3: >66%), lobular inflammation (L; 0: none, 1: <2 sites, 2: 2–4 sites, and 3: >4 sites), and hepatocellular ballooning (B; 0: none, 1: few, and 2: many) [21]. The maximum possible score is 8 points. The pathologist was blinded to the results of speculum examinations.

### 2.3. Hepatic WIRI Model

**Figure 2:**
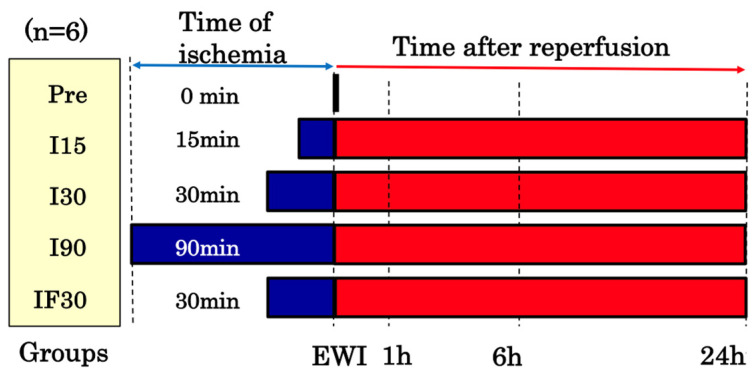
Warm ischemia-reperfusion model time course. The blood flow in the left and middle lobes was blocked, and reperfusion was performed after ischemia. Groups: Pre (immediately after laparotomy), I15 (15 min ischemia group), I30 (30 min ischemia group), I90 (90 min ischemia group), IF30 (30 min ischemia group for fatty livers). EWI: end of warm ischemia.

The rats were anesthetized with isoflurane and, intraoperatively, maintained under inhalational anesthesia with air (2–2.5 mL/min) and isoflurane (1–2.5%). We performed the surgery while using light to keep the rats warm and prevent their body temperature from dropping. The left branches of the portal vein and hepatic artery were blocked with a cerebral aneurysm clip; 70% of the liver was also blocked (Figure 2). The abdomen was closed and kept warm under general anesthesia. The clips were removed, and blood flow was resumed at 15, 30, or 90 min after the initiation of blood flow ischemia in rats with a normal liver and at 30 min after the initiation of blood flow ischemia in rats with fatty liver.

The animals were sacrificed at the end of ischemia and 1, 6, and 24 h after reperfusion. Thereafter, the blood and liver samples were analyzed.

### 2.4. Experimental Groups

Based on when the values were recorded, 108 rats were randomly divided into the following 18 groups, each consisting of 6 rats:

(1) Pre group (n = 6): values recorded immediately after laparotomy when the normal liver was not subjected to ischemia.
(2) EWI15 (n = 6), EWI30 (n = 6), and EWI90 (n = 6) groups: values recorded at the end of warm ischemia (EWI) in normal livers at 15, 30, and 90 min, respectively.
(3) I15/R1h (n = 6), I15/R6h (n = 6), and I15/R24h (n = 6) groups: values recorded at 1, 6, and 24 h, respectively, after 15 min of warm ischemia-reperfusion in normal livers (ischemia/reperfusion); I30/R1h (n = 6), I30/R6h (n = 6), and I30/R24h (n = 6) groups: values recorded at 1, 6, and 24 h, respectively, after 30 min of warm ischemia-reperfusion; and I90/R1h (n = 6), I90/R6h (n = 6), and I90/R24h (n = 6) groups: values recorded at 1, 6, and 24 h after 90 min of warm ischemia-reperfusion, respectively.
(4) PreF group (n = 6): values recorded immediately after laparotomy without exposure to ischemia in fatty livers.
(5) EWIF30 group (n = 6): values recorded at the end of 30 min of warm ischemia in fatty livers.
(6) IF30/R1h (n = 6), IF30/R6h (n = 6), and IF30/R24h (n = 6) groups: values recorded at 1, 6, and 24 h, respectively, after 30 min of warm ischemia-reperfusion in fatty livers.

### 2.5. Sample Collection

At the time of sacrifice, blood was collected from the inferior vena cava, and the liver was removed immediately. The samples were fixed in formalin at room temperature for 24 h and then embedded in paraffin. Samples for frozen sections were embedded in an optimal cutting temperature compound, and samples for IMS were frozen at −80 °C.

### 2.6. Alanine Aminotransferase Activity

The serum alanine aminotransferase (ALT) activity was determined by measuring serum ALT levels with an automated analyzer (AU5400, Beckman Coulter, Brea, CA, USA).

### 2.7. Histopathological Evaluation

Hematoxylin and eosin (HE) staining was performed as described previously. HE-stained specimens were evaluated using the Suzuki injury score for congestion, vacuolation, and necrosis; each parameter was graded on a scale of 0–4. The total score was then compared among the experimental groups. The pathologist was blinded to the results of speculum examinations.

### 2.8. LPO Evaluation 

Malondialdehyde (MDA)+4-hydroxy-2-nonenal (4-HNE) was measured in the liver tissue using the LPO 586 kit (Oxis International, Foster City, CA, USA) in accordance with the manufacturer’s instructions. Frozen liver tissue was homogenized in Tris-HCl (20 mM) containing butylated hydroxytoluene (0.05%), and the homogenate was centrifuged (3000× *g*, 10 min, 4 °C). The supernatant was incubated with the reagent and centrifuged again; the absorbance of the supernatant was measured at 586 nm. A 4-HNE standard was used to prepare a calibration curve, and the results were expressed as the nmol 4-HNE equivalent per mg of wet tissue weight. The protein concentrations were determined using the Pierce BCA Protein Assay Kit (Thermo Fisher Scientific, Waltham, MA, USA).

### 2.9. Evaluation Using IMS

Frozen sections were thinly sliced (10 μm) and attached to indium tin-oxide-coated glass slides (Matsunami Glass Co., Ltd., Osaka, Japan). After spraying the sections with a matrix (9-aminoacridine, 5 mg/mL; 70% EtOH; ACROS, Morris Plains, NJ, USA), the tissue was irradiated with a laser at intervals of 200 μm/pixel or 20 μm/pixel. The sections were then analyzed using MS with a solariX XR system (Bruker Daltonics, Billerica, MA, USA) in negative ion mode to acquire mass spectrum data at *m*/*z* 200–2000.

The matrix application conditions were as follows: TM-Sprayer M3 (HTX Technologies, Chapel Hill, NC, USA), 18 passes; flow rate, 0.1 mL/min; temperature, 75 °C; track spacing, 2 mm; pressure, 10 psi; gas flow, 2 L/min; and velocity, 1200 mm/min.

A total of 14 samples (13 samples from each group plus the internal standard) were placed on a glass slide for simultaneous measurements. A normal liver sample was used as the internal standard and attached to each slide. To compare normal and fatty livers, 11 samples from each 30 min ischemia group and the internal standard were measured simultaneously.

The obtained data were standardized using the total ion current to correct for variations in the ionization efficiency among the samples; the data were corrected for each measurement by expressing them as a ratio to the value of the internal standard. Furthermore, signal ratios were calculated by standardizing a specific molecular signal among the molecules to be compared [22].

### 2.10. Data Processing

The mass spectra obtained immediately after laparotomy (Pre) and at 15, 30, and 90 min of ischemia, as well as at EWI and 1, 6, and 24 h of warm reperfusion, were analyzed using flexImaging^®^ (Bruker Daltonics, Billerica, MA, USA). Signal intensities at each *m*/*z* (tolerance: exact mass *m*/*z* ± 0.05 Da) were calculated and quantified using SCiLS lab 2016a^®^ (Bruker Daltonics).

### 2.11. Molecular Identification

Molecules were identified from the fragmentation pattern of the target molecule following two laser irradiations after matrix application, as in the IMS measurement (multistep mass spectrometry (MS/MS)).

### 2.12. Statistical Analysis

Values are presented as mean ± standard deviation. Comparisons between two groups were performed using Student’s *t*-test, and comparisons between multiple groups were performed using the Tukey–Kramer test. Results with *p* < 0.05 were considered statistically significant. All statistical analyses were conducted using JMP Pro ver. 14.0 for Macintosh (SAS Institute, Inc., Cary, NC, USA).

## 3. Results

### 3.1. Fatty Liver Assessment

**Figure 3:**
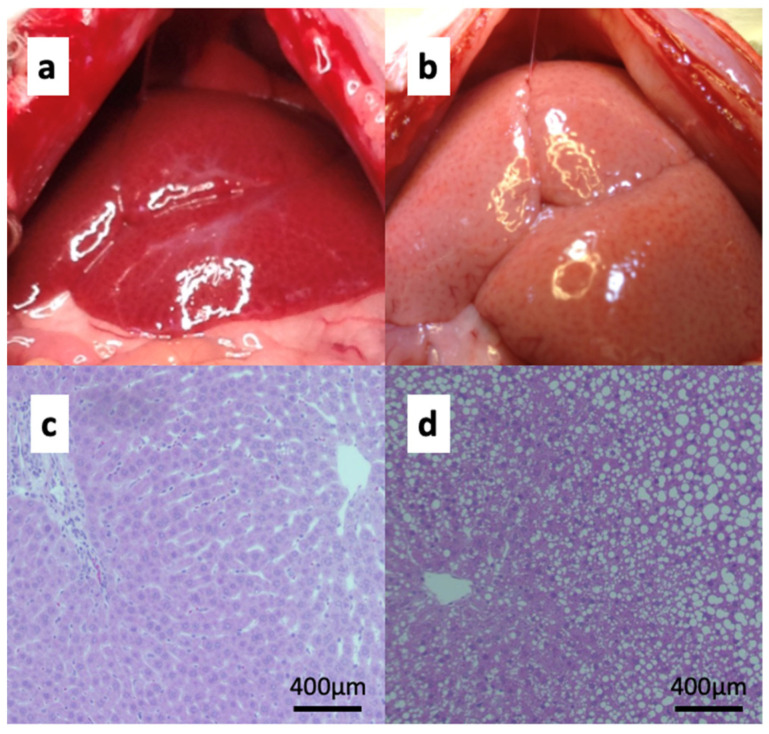
Preparation of the fatty liver (**a**) and normal liver (**b**) at laparotomy. Hematoxylin and eosin (HE) staining (10×) of the normal liver (**c**) and fatty liver (**d**).

The mean NAS for fatty livers was 2.3 ± 0.5 points (S: 2.3 points, L: 0 points, and B: 0 points), and the NAS for normal livers was 0 points (Figure 3).

### 3.2. Validity of the Model

All rats were alive on Postoperative Day 7, with confirmed survival in the 90 min warm ischemia (which showed the highest degree of injury) and 30 min fatty liver ischemia groups.

### 3.3. Serum ALT Activity

The serum ALT level was 42.7 ± 7.7 IU/L in the Pre group. Peak injury was observed at 1 or 6 h after warm ischemia-reperfusion. The serum ALT levels were 362.8 ± 89.3 (*p* < 0.0001), 840.7 ± 328.1 (*p* < 0.0001), and 1830.3 ± 396.4 IU/L (*p* < 0.0001) in the I15/R1h, I30/R1h, and I90/R1h groups, respectively.

**Figure 4:**
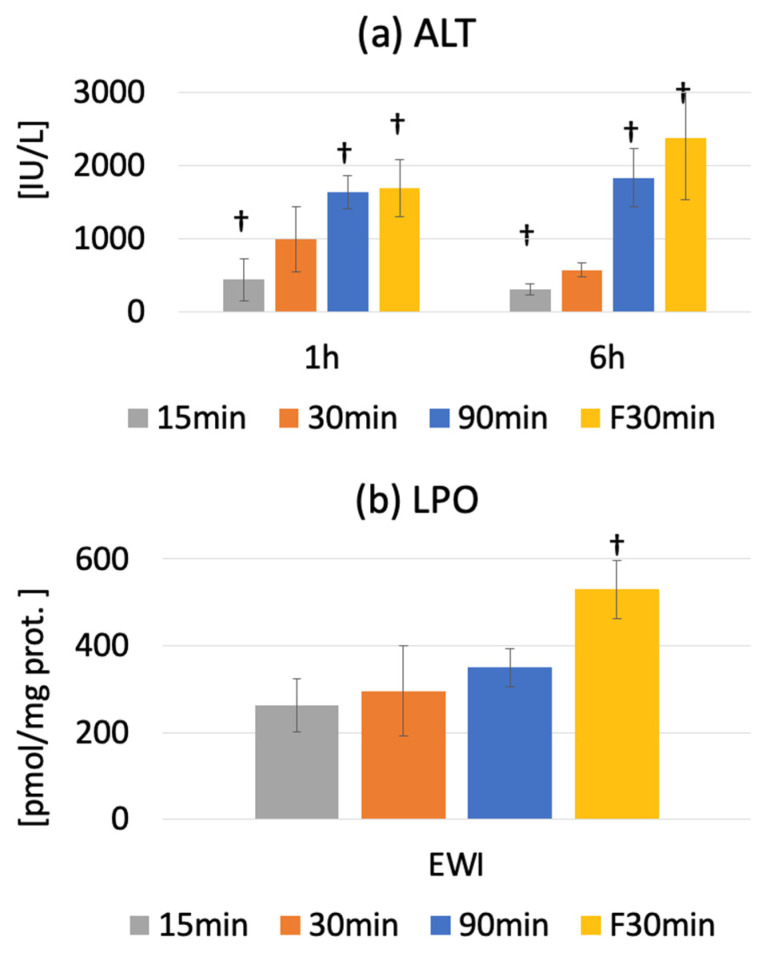
(**a**) Serum alanine aminotransferase (ALT) levels predominantly increased in rats with normal livers in an ischemia time-dependent manner; ALT levels were the highest in rats with fatty livers († *p* < 0.05 vs. the 30 min ischemia group (I30)). (**b**) Hepatic LPO products at the end of warm ischemia were evaluated with the LPO 586 kit (Oxis International, Foster City, CA, USA) using 4-hydroxy-2-nonenal as the standard for a calibration curve. The results showed an increase in the product concentrations at the end of ischemia. There was no significant temporal difference in LPO among normal livers; however, LPO increased in fatty livers with time († *p* < 0.05 vs. the 30 min ischemia group (I30)).

The serum ALT level significantly increased from 67.5 ± 51.0 IU/L (PreF) to 1683.3 ± 392.2 IU/L and 2191.2 ± 683.6 IU/L in the IF30/R1h and IF30/R6h groups, respectively (*p* < 0.0001). Compared to fatty and normal livers, the ALT level was significantly lower in the I30/R1h group than in the IF30/R1h group (*p* < 0.0001); it was also significantly lower in the I30/R6h group than in the IF30/R6h group (*p* < 0.0001). However, the ALT level in the fatty liver was significantly higher in the IF30/R6h group than in the I30/R6h group (*p* < 0.0001). The ALT level decreased at R6h in the I15 and I30 groups but not in the I90 and IF30 groups; this difference widened over time (Figure 4a).

### 3.4. Oxidative Stress

The LPO of MDA+4-HNE in the liver tissue increased significantly at the end of ischemia but decreased from before ischemia to after reperfusion. Additionally, LPO showed an increasing trend along with the ischemia duration; however, the change was not significant.

At the end of ischemia, LPO was significantly higher in the EWIF30 group than in the PreF group (529.7 ± 45.4 vs. 314.8 ± 46.8 nmol/mg protein, *p* < 0.0001), indicating higher oxidative stress in fatty livers than in normal livers (*p* < 0.0001) (Figure 4b).

### 3.5. Suzuki Injury Score

**Figure 5:**
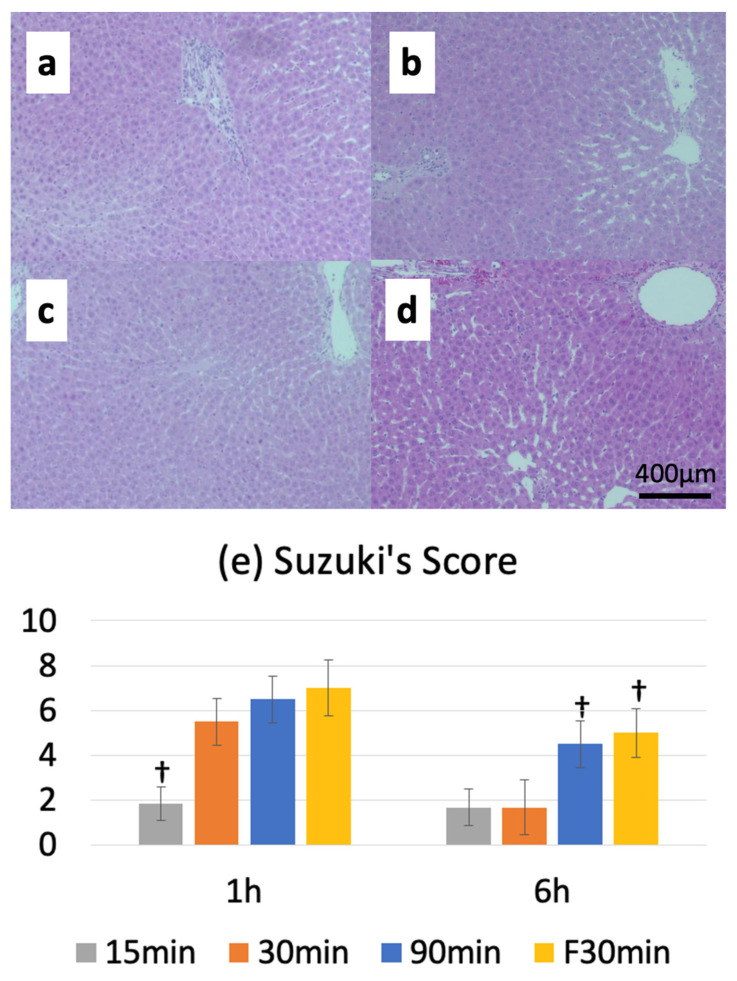
Hepatocellular injury, sinusoidal congestion, and ballooning of hematoxylin and eosin (HE)-stained specimens were graded on a 0–3 scale, and the total scores were compared. The 30 min ischemia (IF30) group for fatty livers showed the same injury as the 90 min warm ischemia (I90) group for normal livers; neither group recovered from the injury at 6 h after reperfusion (R6h). (**a**–**d**) HE staining: (**a**) Pre (immediately after laparotomy, when the normal liver was not subjected to ischemia), (**b**) I15 (15 min of warm ischemia)/R6h, (**c**) I30 (30 min of warm ischemia)/R6h, and (**d**) I90 (90 min of warm ischemia)/R6h). (**e**) Graphical representation of the Suzuki injury score († *p* < 0.05 vs. the 30 min ischemia group (I30)).

The Suzuki injury score before ischemia was 0, and the reperfusion injury score peaked at 1 or 6 h after warm ischemia-reperfusion. The I15/R1h, I30/R1h, and I90/R1h groups had Suzuki injury scores of 2.0 ± 0.6, 5.5 ± 1.0, and 6.5 ± 1.0, respectively. Furthermore, the Suzuki injury score was 7.0 ± 1.3 in the IF30/R1h group. A comparison between normal and fatty livers revealed no significant differences in the Suzuki injury scores between the I30/R1h (5.5 ± 1.0) and IF30/R1h (7.0 ± 1.3) groups; however, a significant difference was observed in the Suzuki injury scores between the I30/R6h (1.7 ± 1.2) and IF30/R6h (5.0 ± 1.1) groups (Figure 5a–e).

### 3.6. IMS

**Figure 6:**
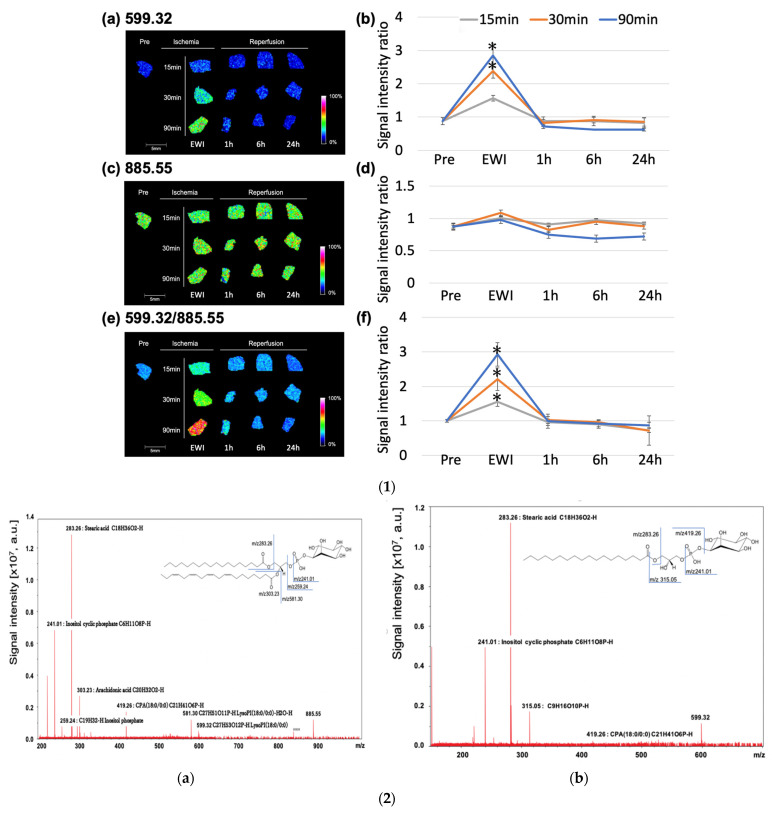

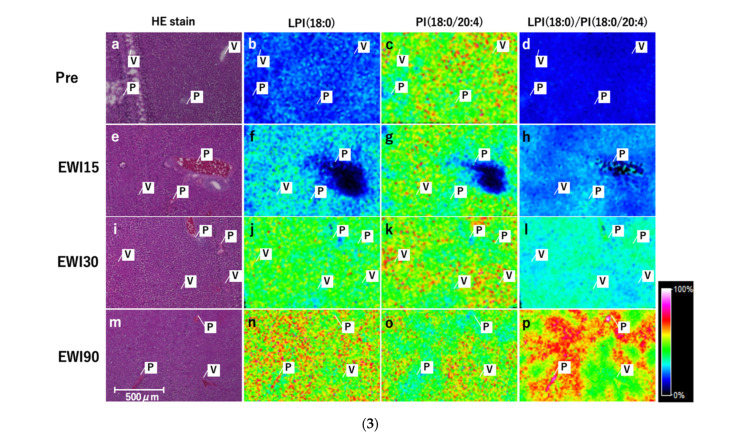
(**1**) Ischemia duration and molecular changes after reperfusion in normal livers. We established a rat model with 70% ischemia-reperfusion in a normal liver and compared the outcomes at different ischemia durations. The ischemia durations were 15, 30, and 90 min, and samples were collected at 1, 6, and 24 h after reperfusion. Organ sections were comprehensively analyzed using imaging mass spectrometry for molecules with *m*/*z* 200–2000 (* *p* < 0.05 vs. Pre group (immediately after laparotomy, when the normal liver was not subjected to ischemia). (**a**,**b**) *m*/*z* 599.32; (**c**,**d**) *m*/*z* 885.55; (**e**,**f**) *m*/*z* 599.32/*m*/*z* 885.55; (**a**,**c**,**e**) IMS results; (**b**,**d**,**f**) signal intensity ratios. The *m*/*z* 599.32 increased at the end of ischemia, and *m*/*z* 599.32/*m*/*z* 885.55 increased in a more ischemic time-dependent manner. (**2**) (**a**) *m*/*z* 885.54 was subjected to multistep mass spectrometry. The phosphatidylinositol (PI) headgroup (*m*/*z* 241.01, *m*/*z* 259.24), stearic acid (18:0) (*m*/*z* 283.26), arachidonic acid (20:4) (*m*/*z* 303.23), cyclic phosphatidic acid (CPA) (18:0) (*m*/*z* 419.26), and lysophosphatidylinositol (LPI) (18:0) (*m*/*z* 581.30, *m*/*z* 599.32) were detected and identified as PI (18:0/20:4). (**b**). *m*/*z* 599.32 was determined using multistep mass spectrometry. The phosphatidylinositol (PI) headgroup (*m*/*z* 241.01, *m*/*z* 315.05), stearic acid (18:0) (*m*/*z* 283.26), and cyclic phosphatidic acid (CPA) (18:0) (*m*/*z* 419.26) were detected and identified as lysophosphatidylinositol (LPI (18:0)). (**3**) Localization changes in normal livers with the ischemia duration (before and after 15, 30, and 90 min of ischemia). (**a**,**e**,**i**,**m**) Hematoxylin and eosin staining. (**b**,**f**,**j**,**n**) Lysophosphatidylinositol (LPI (18:0)) localization. (**c**,**g**,**k**,**o**) Phosphatidylinositol (PI (18:0/20:4)) localization. (**d**,**h**,**l**,**p**) LPI (18:0)/PI (18:0/20) localization. (**a**–**d**) Pre-ischemia (Pre). (**e**–**h**) At the end of 15 min of ischemia (EWI15). (**i**–**l**) At the end of 30 min of ischemia (EWI30). (**m**–**p**) At the end of 90 min of ischemia (EWI90). P: portal vein (Glisson sheath). V: central venous area. LPI (18:0)/PI (18:0/20:4) changed from “high” in Zone 1 to “low” in Zone 3 and showed a clear maldistribution with increased ischemia duration.

The *m*/*z* 599.32 (lysophosphatidylinositol (LPI (18:0))) peaked at the end of ischemia in each group (Figure 6(1a,b)). Compared to the Pre group, the *m*/*z* 599.32 increased significantly with ischemia duration after 30 min (EWI15: 1.56 ± 0.2, *p* = 0.1479, EWI30: 2.39 ± 0.53, *p* = 0.0004, and EWI90: 2.85 ± 0.85, *p* < 0.0001). There were no significant differences after reperfusion.

There was no significant difference in the *m*/*z* 885.55 among the evaluated time points (phosphatidylinositol (PI (18:0/20:4))) when compared to the Pre group; however, after reperfusion, the *m*/*z* 885.55 decreased with the ischemia duration, with the greatest decrease occurring 1 h after reperfusion after 15 and 30 min of ischemia (I15/R1h: 0.90 ± 0.25, I30/R1h: 0.82 ± 0.31, and I90/R6h: 0.68 ± 0.09; Figure 6(1c,d)). Additionally, the *m*/*z* 885.55 in the I90/R6h group showed the greatest decrease.

The *m*/*z* 599.32/*m*/*z* 885.55 (LPI (18:0)/PI (18:0/20:4) peaked at the end of ischemia in each group; compared to its value of 1.01 ± 0.04 in the Pre group, the value increased significantly in all ischemia groups (EWI15: 1.56 ± 0.14, *p* = 0.0045, EWI30: 2.21 ± 0.33, *p* < 0.0001, and EWI90: 2.92 ± 0.34, *p* < 0.0001). There was a significant difference depending on the ischemia duration; however, no differences were noted after reperfusion (Figure 6(1e,f)).

### 3.7. Molecular Identification

Multistep MS of the molecular species at *m*/*z* 885.54 is shown in Figure 6(2a).

The PI headgroup (*m*/*z* 241.01, *m*/*z* 259.24), stearic acid (18:0; *m*/*z* 283.26), arachidonic acid (20:4; *m*/*z* 303.23), cyclic phosphatidic acid (CPA; 18:0; *m*/*z* 419.26), and LPI (18:0; *m*/*z* 581.30, *m*/*z* 599.32) were detected and identified as PI (18:0/20:4).

### 3.8. Multistep MS of the Molecular Species at m/z 599.32

The PI headgroup (*m*/*z* 241.01, *m*/*z* 315.05), stearic acid (18:0; *m*/*z* 283.26), and CPA (18:0; *m*/*z* 419.26) were detected and identified as LPI (18:0).

### 3.9. Localization Analysis of LPI and PI

There was no change in the signal in the Pre group with respect to LPI (18:0); however, the overall signal increased with the ischemia duration, with a relatively strong signal obtained around the portal vein (Zone 1) and a relatively weak signal around the central vein (Zone 3). The overall signal for PI (18:0/20:4) did not differ among any of the evaluated ischemia time points; however, the signal changed from “low” in Zone 1 to “high” in Zone 3. In LPI (18:0)/PI (18:0/20:4) as in LPI (18:0), the overall signal increased with the ischemia duration, was higher in Zone 1 and lower in Zone 3, and showed a clear maldistribution with the ischemia duration (Figure 6(3a–p)).

### 3.10. Comparison between Normal and Fatty Livers

Compared to groups before ischemia, the *m*/*z* 599.32 (LPI (18:0)) had significantly increased, and the signal intensity had peaked at the end of ischemia in the normal and fatty liver groups (1.29 ± 0.56 vs. 0.92 ± 0.18). The signal intensity was significantly higher only at the end of ischemia in the normal liver group (EWI30: 3.93 ± 1.1; EWIF30: 1.94 ± 1.0). However, compared to the fatty liver group, the signal intensities were higher at all time points in the normal liver group; only EWI showed a significant difference (EWI30: 3.93 ± 1.1 vs. EWIF30: 1.94 ± 1.0).

There was no significant change in the *m*/*z* 885.55 (PI (18:0/20:4)) with reperfusion in the normal and fatty liver groups; however, the signal intensity was higher in normal livers than in fatty livers at all time points. Additionally, it was significantly higher at EWI and 1, 6, and 24 h after reperfusion.

**Figure 7:**
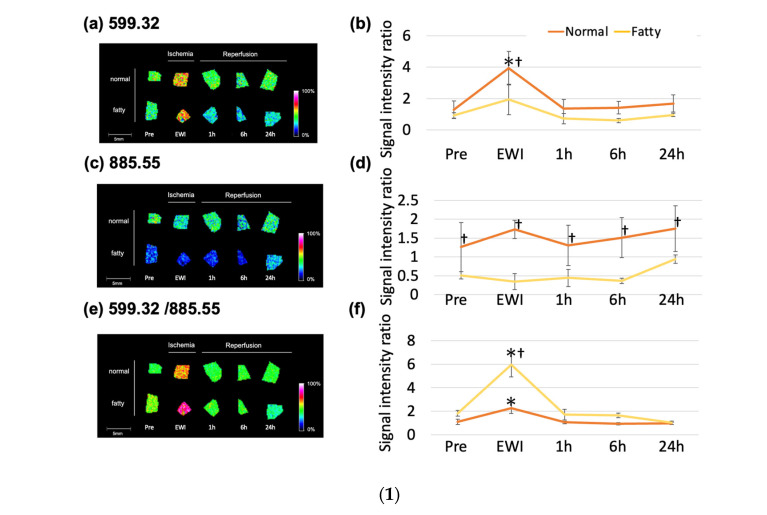

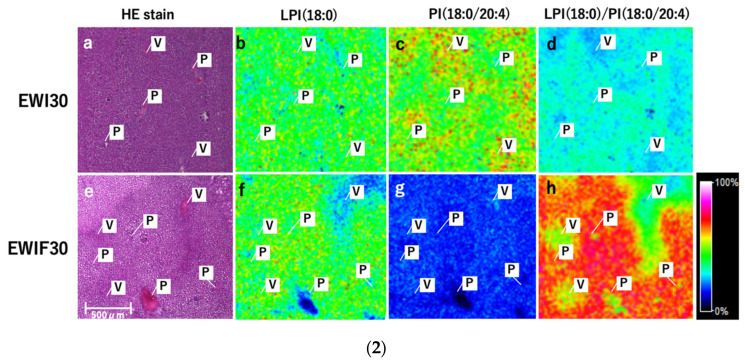
(**1**) Comparison of normal and fatty livers and molecular changes after 30 min of inhibition-reperfusion. A rat model with 70% inhibition-reperfusion was constructed, and samples were collected at 1, 6, and 24 h after 30 min of inhibition-reperfusion. The organ sections were comprehensively analyzed using imaging mass spectrometry (IMS) for molecules with *m*/*z* 200–2000, and normal and fatty livers were compared (* *p* < 0.05 vs. the Pre group (immediately after laparotomy, when the normal liver was not subjected to ischemia), † *p* < 0.05 for comparison of normal and fatty livers at the same time point). (**a**,**b**) *m*/*z* 599.32. (**c**,**d**) *m*/*z* 885.55. (**e**,**f**) *m*/*z* 599.32/*m*/*z* 885.55. (**a**,**c**,**e**) IMS results. (**b**,**d**,**f**) Signal intensity ratios for fatty liver phosphatidylinositol (18:0/20:4) were decreased in fatty livers, but the *m*/*z* 599.32/*m*/*z* 885.55 ratios were higher in fatty livers than in normal livers. (**2**) Localization changes in normal and fatty livers at the end of 30 min of inhibition: (**a**,**e**) hematoxylin and eosin staining; (**b**,**f**) localization of lysophosphatidylinositol (LPI (18:0)); (**c**,**g**) localization of phosphatidylinositol (PI (18:0/20:4)); (**d**,**h**) localization of LPI (18:0)/PI (18:0/20:4); (**a**–**d**) normal liver at the end of 30 min of inhibition (EWI30); (**e**–**h**) fatty liver at the end of 30 min of inhibition (EWIF 30). P: portal vein (Gleason sheath). V: central venous area LPI (18:0). LPI (18:0)/PI (18:0/20:4) showed clear maldistribution with an enhanced signal in Zone 1 (as in normal livers).

The *m*/*z* 599.32/*m*/*z* 885.55 (LPI (18:0)/PI (18:0/20:4) was significantly higher in the Pre group (1.01 ± 0.23) than in the PreF (1.83 ± 0.24) and EWIF30 (5.95 ± 1.0) groups (Figure 7(1)). In addition, it was significantly higher in the fatty liver group than in the normal liver at EWI.

### 3.11. Comparison between Normal and Fatty Livers

LPI (18:0) had a stronger signal in Zone 1 and a weaker signal in Zone 3 in fatty and normal livers (Figure 7(2b,f)). PI (18:0/20:4) had a weaker signal in Zone 1 and a stronger signal in Zone 3 (Figure 7(2c,g)), and the overall signal was markedly decreased in fatty livers. For LPI (18:0)/PI (18:0/20:4), the signal was stronger in Zone 1 and weaker in Zone 3. These changes were more prominent in fatty livers (Figure 7(1a–f,2a–h)).

## 4. Discussion

We hypothesized that low-molecular-weight compounds, including lipids and fatty acids, are responsible for the development of reperfusion injury during warm hepatic ischemia and LPI (18:0) and PI (18:0/20:4) are the candidate key players in this process. We have discovered that the initial conversion of PI (18:0/20:4) to LPI (18:0) in Zone 1 during hepatic warm ischemia is influenced by the duration of ischemia and is more noticeable in fatty livers. The rise in LPI (18:0) towards the end of ischemia has an impact on the outcomes of post-reperfusion injury. The ratio of LPI (18:0)/PI (18:0/20:4) could serve as a potential predictive marker for post-reperfusion injury.

Our warm ischemia-reperfusion model was non-lethal in the normal liver 90 min and fatty liver ischemia groups, and it could be evaluated without secondary liver injury due to unrelated factors such as circulatory and respiratory failure. The blood ALT activity increased at R1h in normal livers in an ischemia-duration-dependent manner; it decreased at R6h in the group with less than 30 min of ischemia but did not recover in the I90 group, consistent with the findings of a previous report [23]. ALT activity in fatty livers was similar to that in the I90/R1h group; however, it surpassed that in the I90/R6h group. The Suzuki injury score also showed time-dependent injury and exacerbation in fatty livers. Additionally, recovery from injury was not possible when the ischemia duration exceeded a threshold value, suggesting that this assessment method could predict differences in the quality of the stress response after reperfusion. However, these measures are not predictive markers of injury, as they did not change significantly during ischemia; thus, identifying new markers is warranted. In the present study, the MDA+4-HNE level (as a measure of LPO) was markedly increased during ischemia in fatty livers; however, it did not reflect injury associated with ischemia duration in normal livers. The initial reaction products of LPO, such as phosphatidylcholine hydroperoxide (PC-OOH) and phosphatidylethanolamine hydroperoxide (PE-OOH), increased in inhibited blood in a time-dependent manner. Although these products can be used to predict injury after reperfusion, they are unsuitable for clinical application because of technical difficulties concerning autoxidation and degradation during the extraction and preparation of standards. However, when discussing LPO in terms of MDA and 4-HNE, it should be noted that the reaction that generates MDA and 4-HNE from PC-OOH and PE-OOH does not proceed well under hypoxia; this may lead to an underestimation of the oxidative stress in the inhibited blood [12]. 

Herein, we used IMS to solve these problems because it is not only a simple method to comprehensively search for variable molecules but its results have enhanced biological significance as they are unaffected by artifacts arising from sample manipulation (such as extraction, concentration, and drying). In fact, molecules identified using IMS showed various trends in changes during and after WIRI, and molecules that sensitively reflected the effect of ischemia time were identified. Because the signal intensity varies with the thickness of the matrix, it is recommended to use a dedicated device for spraying, as in our study.

LPI (18:0) is a lysophospholipid in which the side chain fatty acid of PIs with stearic acid (FA18:0), including phospholipid PI (18:0/20:4), is dropped out. Although the overall fatty acid composition or the fatty acid composition of the side chain of phospholipids varies depending on the organ and cell type, the fatty acid composition of the side chain of PI in the rat liver is 36.4% FA18:0 and 51.3% FA20:4 [24]. Because cytosolic phospholipase A2α, which shows high substrate selectivity for arachidonic acid-containing phospholipids, is involved in the release of arachidonic acid (FA20:4), it may be one of the main pathways for LPI (18:0) formation [25]. Furthermore, in our study, the LPI (18:0)/PI (18:0/20:4) ratio was significantly increased in the whole liver at the end of ischemia (even after a short period of ischemia), especially in Zone 1. Thus, we infer that this ratio can be a sensitive indicator of the change from PI (18:0/20:4) to LPI (18:0) in the liver (especially in Zone 1). However, whether LPI (18:0) is a cause of enhanced reperfusion injury or a hepatoprotective stress response remains unclear. Furthermore, the association between an increase in LPI (18:0) and the actual intensity of the injury and prognosis remains unclear.

As in normal livers, LPI (18:0) increased at the end of ischemia in fatty livers; however, the corresponding signal intensity was weaker in fatty livers than in normal livers. This difference was probably due to the lower amounts of PI (18:0/20:4), which is the source of LPI (18:0), in fatty livers. The LPI (18:0)/PI (18:0/20:4) ratio at the end of ischemia was significantly higher in fatty livers than in normal livers; this was also observed in normal livers with 90 min of ischemia. Thus, the LPI (18:0)/PI (18:0/20:4) ratio (derived from PI (18:0/20:4)) may be a better indicator of stress. However, the behavior of lipids and fatty acids may differ depending on fatty liver models and species. In this regard, a limitation of our study was that the findings of our rat model might be species-specific and, therefore, developing and using models whose results may have better applicability to humans is necessary.

In the present study, LPI-specific quantitative and localization changes were observed, but the effects of LPI on hepatic WIRI remained unelucidated. The biological activities of LPI were reported recently. The role of the LPI-ligand receptor, G-protein-coupled receptor 55 (GPR55) [26], is that LPI regulates immune cell function [27], is expressed in the liver [28], and is present in elevated levels in the damaged livers of humans and animals [29]; its levels also increase in hepatic warm ischemia. LPI may also play a role in the development of WIRI via GPR55. The Rho/ROCK pathway, which acts upstream of GPR55, exacerbates injury in myocardial ischemia-reperfusion [30], while ROCK ischemia suppresses injury in hepatic ischemia-reperfusion [13]. Based on these reports and the findings of the present study, we infer that the LPI–GPR55 pathway may be involved in the effector mechanism leading to injury and may be a highly selective therapeutic target. However, the mechanism of GPR55-mediated injury in both parenchymal and non-parenchymal cells in hepatic hyperthermia-reperfusion is unknown and should be investigated in future studies.

Hepatic WIRI is more pronounced in Zone 3 (the central venous region); the oxygen concentration in Zone 3 is lower than that in Zone 1 (the portal venous region), wherein the oxygen concentration is higher in the sinusoids [31]. In the present study, LPI (18:0) increased in Zone 1. Just as LPI enhances ischemia-reperfusion injury in the myocardium, it may also be involved in and enhance hepatic injury. Notably, it would be compatible with zoning since LPI is higher in Zone 1 and lower in Zone 3, where the injury is more severe in the liver. However, this hypothesis needs to be tested in the future. 

Nevertheless, we were able to evaluate the changes in low-molecular-weight compounds along with zonation (Zones 1–3), which is a unique result of the IMS-based analyses of our study. In the future, understanding the causes of changes in these low-molecular-weight compounds and the downstream biological reactions at the protein and epigenetic levels will help in elucidating pathological conditions and may lead to the discovery of new markers and therapeutic targets.

LPI (18:0) fluctuates dramatically during warm ischemia prior to cell death, reflecting the time of warm ischemia. It is relatively stable and can exist in both lipid-soluble and water-soluble fractions; thus, it may leak into the blood from open hepatic veins during warm ischemia. Therefore, it is possible to detect hepatic hemostatic changes in the blood during warm ischemia; in fact, LPI quantification in human blood using liquid chromatography–MS has already been reported [32]. Additionally, because LPI is produced through an enzymatic reaction using PI (18:0/20:4) as a substrate, it is unlikely to cause artifacts (unlike in non-enzymatic reactions), thus serving as a promising prognostic marker for IRI.

## 5. Conclusions

Hepatic warm ischemia induces the initial conversion of PI (18:0/20:4) to LPI (18:0) in Zone 1 in an ischemia-duration-dependent manner. This change is more pronounced in fatty livers. The increase in LPI (18:0) at the end of ischemia affects post-reperfusion injury outcomes. The LPI (18:0)/PI (18:0/20:4) ratio may be a potential predictive marker for post-reperfusion injury.

## Data Availability

The data presented in this study are available on request from the corresponding author. The data are not publicly available due to some ongoing analyses.
